# A Toothless People

**Published:** 1875-02

**Authors:** 


*A Toothless People.*—Under this title the following appears in some of our medical exchanges, which, if part at least be true, is a good lesson to those who require to become wiser through tribulation. We have very little sympathy for such people, when we know that in and about Warrenton, Va., there are several excellent practitioners of Dentistry.:

"A few weeks ago a dentist came to town and advertised that he would 'remove all of a person's teeth for two dollars and insert a new set for ten dollars, besides giving six months' credit.' The Warrenton people are very fond of bargains, so there was a rush for the dentist's office. He was busy for two weeks pulling teeth, and at the end of that time half the people had empty gums, and a bone-dust factory in the neighborhood doubled its workmen so as to grind up the teeth.

"Meanwhile the people were waiting for the dentist to fit them with new sets. The abandoned scoundrel eloped with the hotel keeper's wife, and now there are two or three thousand people in town who cannot eat anything tougher than soup and farina. All the butchers have failed, and not a cracker has been sold for three weeks. One man, it is said, whittled out a set of wooden teeth for himself, but the first drink of whiskey he took—Cincinnati whiskey—set them in a blaze, and his funeral came off next day. The dentist will hear of something to his disadvantage if he comes back."

